# Optical Design with Narrow-Band Imaging for a Capsule Endoscope

**DOI:** 10.1155/2018/5830759

**Published:** 2018-01-10

**Authors:** Chih-Ta Yen, Zong-Wei Lai, Yu-Ting Lin, Hsu-Chih Cheng

**Affiliations:** ^1^Department of Electrical Engineering, National Formosa University, Yunlin, Taiwan; ^2^Department of Electro-Optics Engineering, National Formosa University, Yunlin, Taiwan

## Abstract

The study proposes narrow-band imaging (NBI) lens design of 415 nm and 540 nm of a capsule endoscope (CE). The researches show that in terms of the rate of accuracy in detecting and screening neoplastic and nonneoplastic intestinal lesions, the NBI system outperformed that of traditional endoscopes and rivaled that of chromoendoscopes. In the proposed NBI CE optical system, the simulation result shows the field of view (FOV) was 109.8°; the modulation transfer function (MTF) could achieve 12.5% at 285 lp/mm and 34.1% at 144 lp/mm. The relative illumination reaches more than 60%, and the system total length was less than 4 mm. Finally, this design provides high-quality images for a 300-megapixel 1/4^″^ CMOS image sensor with a pixel size of 1.75 *μ*m.

## 1. Introduction

Modern-day eating habits have increased the burden on the digestive systems of most people, and gastrointestinal diseases have emerged as a widespread problem. Some of the conventional gastrointestinal examination methods include gastroscopy and colonoscopy. The former examines areas such as the esophagus, stomach, duodenum, and the front section of the small intestine, whereas the latter (in which the examination device is inserted through the anus) examines areas such as the rectum, the large intestine, and the end section of the small intestine. The small intestine, which makes up 75% of the human gastrointestinal tract, is the site where symptoms are most difficult to spot. Because conventional endoscopes are invasive, most people fear to visit the hospital; as a result, their diseases worsen. However, with advances in medical technology in recent years, a new tool has been developed to inspect intestinal diseases; it is called the “capsule endoscope” (CE) [1].

After the patient has swallowed a CE, it is pushed through the patient's digestive tract by the mechanism of enterogastric peristalsis. The CE is equipped with a lighting source, an optical lens, and an image sensor. By using such devices, it captures images of the human digestive organs; image data are subsequently sent through radiofrequency (RF) and microwave signals to an external storage device worn by the patient for data storage. The CE procedure, in which the CE enters from the esophagus to the stomach and later to the small intestine, takes approximately six to eight hours to complete. A CE takes color photographs at a rate of two photographs per second, producing roughly 50,000–60,000 photographs during the process. The CE is later excreted naturally, completing the inspection process. Finally, computers are used to read image data extracted from the storage device memory, and data are made into dynamic videos viewed by the physicians who diagnose the diseases. Lewis and Swain indicated that the use of CEs can improve disease detection rate as well as lowering the discomfort experienced by patients [2]. This showed that CE examination is a more accurate and beneficial method for inspecting small intestinal diseases than traditional methods.

In 1999, Given Imaging designed a CE prototype, and in August 2011, it developed an official CE approved by the FDA; the product was subsequently released in the US market. Currently, Olympus is the major domestic CE brand in Japan. The company's patent [3] presents an optical system design similar to that in the study of Ramsden eyepieces [4]. By considering the size limit of the CE, they proposed a single plastic lens with an aspherical surface composed of two flat convex lenses, thus simplifying the structure and meeting the volume limits. This design makes the optical system smaller by reducing the number of lenses and shortening the optical path. However, when the number of lenses is reduced, the quality of the image also decreases and the distortion increases. The lens parameters of the proposed patent are as follows: effective focal length, *f* = 1 mm; field of view angle = 85°–110°; optical total length, *L* < 2.4 mm; image plane radius < 1 mm; and depth of field (DOF) = 5.45–30 mm. Therefore, the lens can be used only on CCD or CMOS image sensors smaller than 1/10^″^. Seo et al. proposed an MEMS-based liquid lens, which can improve image quality by using the autofocus and zoom-in functions. However, a bias voltage of up to 30 V is required to consider the actual characteristics of CE, and hence, the commercialization of CE systems is difficult [5].

Chen [6] noted that the use of a conical optical reflector and a transparent, ring-shaped view window in the CE design served the primary purpose of overcoming CE mechanism limitations. Early CEs demonstrated astigmatism defects in focus. By increasing the number of focal lens groups and changing the location of the conical optical reflector, Chen corrected the astigmatism of the conical optical reflector and enhanced the contact-based CE image quality [6]. In that study, the CE captured images primarily from its sides and had an FOV of 65.08°. The new lens group, which features an F/# of 4.2, generated a maximum MTF of 36% at a spatial frequency of 100 lp/mm.

Ou-Yang and Jeng introduced a new-generation radial imaging capsule endoscope (RICE) that achieved ring-field scanning by using a conical lens; a transparent, ring-shaped view window; and an optical focusing system [7]. Because stray light lowered the lighting uniformity of the RICE, various improvements were made to the RICE lighting system, including changing the locations of LEDs, cutting off a section of the tip of a conical lens, and employing a multilayer coating method, after which lighting uniformity was enhanced from 12% to 69%. In addition, a clinical, in vivo experiment was administered to verify the effectiveness of the RICE. The device was fed to a pig. Small images of the pig's small intestine were stitched together to form composite images. Pearson's correlation coefficients verified that the images effectively represented complex intestinal environments. Tseng et al. [8] proposed a color multiplexing capsule endoscope (CMCE), which adopted a color multiplexing method to enable its x-cube prism to simultaneously record frontal images as well as those on the two sides [8]. The images were presented in three different colors, namely, green, red, and blue. Each side of the CMCE had a viewing angle of 70°, and images were combined to produce a full viewing angle of 210°. This allowed the CE to produce favorable image quality with a miniaturized device.

CEs can be classified into three types. In this study, the traditional frontal image-based CE model was upgraded by using CE structures proposed by Mang et al. [9] and Tang et al. [10]. The former reduced distortion without adding additional lenses (to avoid increasing the size of the CE) by making the CE optical protective mask into a lens and turning object planes into flat planes, whereas the latter used fewer aspheric lens than were used in [9] and enhanced lens groups to facilitate CE production.

## 2. Research Methods

This section presents the overall design process of a CE optical system; optical system design methods can be divided into the following steps: (1) determining the optical uses of the system, (2) setting design indicators, (3) selecting initial structure, (4) correcting optical aberrations, and (5) testing and inspecting performance.

### 2.1. Setting Specifications

Because the CE must travel through human organs, the aperture and effective focal lens (EFL) values of its cameras cannot be overly large. Therefore, this study set the EFL, back focal length (BFL), and F/# at 2 mm, 1 mm, and 2.8, respectively. In addition, because the overall CE size must be controlled, the total length of a camera lens was set at approximately 4 mm.

**Figure 1:**
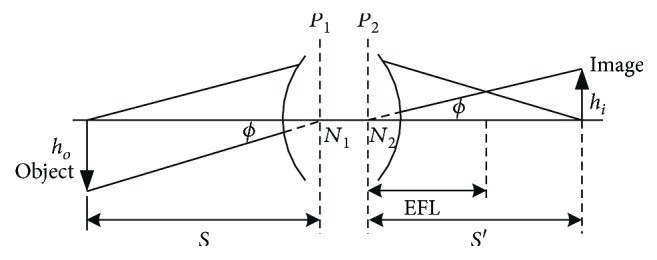
Schematic of the image and object in relation to each other.

The CMOS selected in this study was an OV3640 model, which featured a complementary metal–oxide–semiconductor (CMOS) image sensor, an effective pixel arrangement of 2048 × 1536, a total resolution of approximately 300 million pixels (size of single pixel = 1.75 *μ*m × 1.75 *μ*m), and a semi-image height of 2.3 mm. Using Figure 1, the object distance, image distance, and FOV required were derived.

The Gaussian imaging equation is as follows:

(1) $$\begin{array}{c} \frac{1}{s^{\prime }}-\frac{1}{s}=\frac{1}{f}. \end{array}$$

The image longitudinal magnification equation is as follows:

(2) $$\begin{array}{c} m=\frac{h_{i}}{h_{o}}=\frac{s^{\prime }}{s}. \end{array}$$

By multiplying the two sides of (2) by *s*, the following equation is derived:

(3) $$\begin{array}{c} s=f\left(\frac{1}{m}-1\right), \end{array}$$

where *f*, *s*, and *s*′ are the EFL, object distance, and image distance, respectively.

Because the diameter of the small intestine is approximately 25 mm [11] and the length of an intestinal villus is approximately 1.25 mm, object height was set at approximately 11.25 mm. In addition, because one-half of the length of the diagonal line joining the image height and the object was approximately 2.3 mm, *h*_*o*_ and *h*_*i*_ were set at 11.25 and 2.3 mm, respectively. Using (3), the *m*, *s*, and *s*′ were calculated as 0.2, 8, and 1.6, respectively. The FOV was determined by solving the equations tan*ϕ* = *h*_*o*_/*s* and *ϕ* = tan^−1^(*h*_*o*_/*s*), that is, *ϕ* = 54.6°. Therefore, FOV was calculated to be approximately 109.2°.

The design indicators of this study are shown in Table 1 [12].

### 2.2. Image Sensor

The human body structure was considered for calculating the optical system specifications in the study (Table 1). The CMOS model OV3640 was selected in this study, and its specifications are as follows: an effective pixel arrangement of 2048 × 1536 and a total resolution of approximately 300 million pixels (size of single pixel = 1.75 × 1.75 *μ*m). A comparison of the specifications of different CMOS image sensors used in optical CE systems is presented in Table 2 [13].

When evaluating the quality of a camera lens, the most essential criteria are its resolution and sharpness. At a low spatial frequency, a camera lens that produces high MTF indicates superior contrast, that is, clearer image outline; similarly, at a high spatial frequency, a camera lens that produces high MTF signifies more favorable sharpness, that is, clearer subtle details. The spatial frequency that can be analyzed by a CMOS image sensor is related to the pixel size of the images. By using (4), the Nyquist frequency can be obtained [14].

(4) $$\begin{array}{c} MTF=\frac{1}{2\times pixel\;size\;\left(mm\right)}. \end{array}$$

Given that the size of a single pixel was 1.75 *μ*m, the maximum spatial frequency was calculated to be 285.7 lp/mm. To obtain favorable image quality, the spatial frequency should be 10% or higher at an MTF of 285 lp/mm.

### 2.3. Narrow-Band Imaging (NBI)

**Figure 2:**
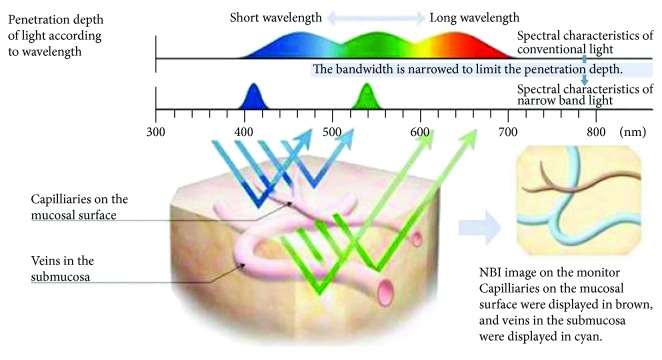
Narrow-band imaging [17].

The accuracy of traditional white-light endoscopy in identifying and diagnosing neoplastic and nonneoplastic lesions is approximately 70%, whereas that of chromoendoscopy and magnifying endoscopy is between 90% and 95%. Because chromoendoscopy uses a photographic developer to highlight subtle lesion changes, it requires dyes to be sprayed in advance, which increases treatment time. Virtual chromoendoscopes have thus been developed. Of the various imaging methods adopted by virtual chromoendoscopes, the narrow-band imaging method is the most common. Narrow-band technology was developed based on a physical theory; that is, the longer the wavelength, the better its penetration rate. In the visible light spectrum, red light wave has the longest wavelength, followed by green and blue light waves. When two light sources of different wavelengths [i.e., blue (wavelength: 415 nm) and green (wavelength: 540 nm)] illuminate the intestine, the blue and green light waves penetrate shallower and deeper layers of the gastrointestinal tract mucosa, respectively (Figure 2). Because capillaries (brown in color) in the epithelial mucosal tissues are detectable by blue light waves and because blood vessels (cyan in color) in the subcutaneous mucosal tissues are detectable by green light waves, they assist observers in identifying epithelial and subcutaneous mucosal tissue lesions. Sano et al. [15] and Machida et al. [16] both showed that in terms of the rate of accuracy in detecting and screening neoplastic and nonneoplastic intestinal lesions, the NBI system outperformed that of traditional endoscopes and rivaled that of chromoendoscopes.

**Figure 3:**
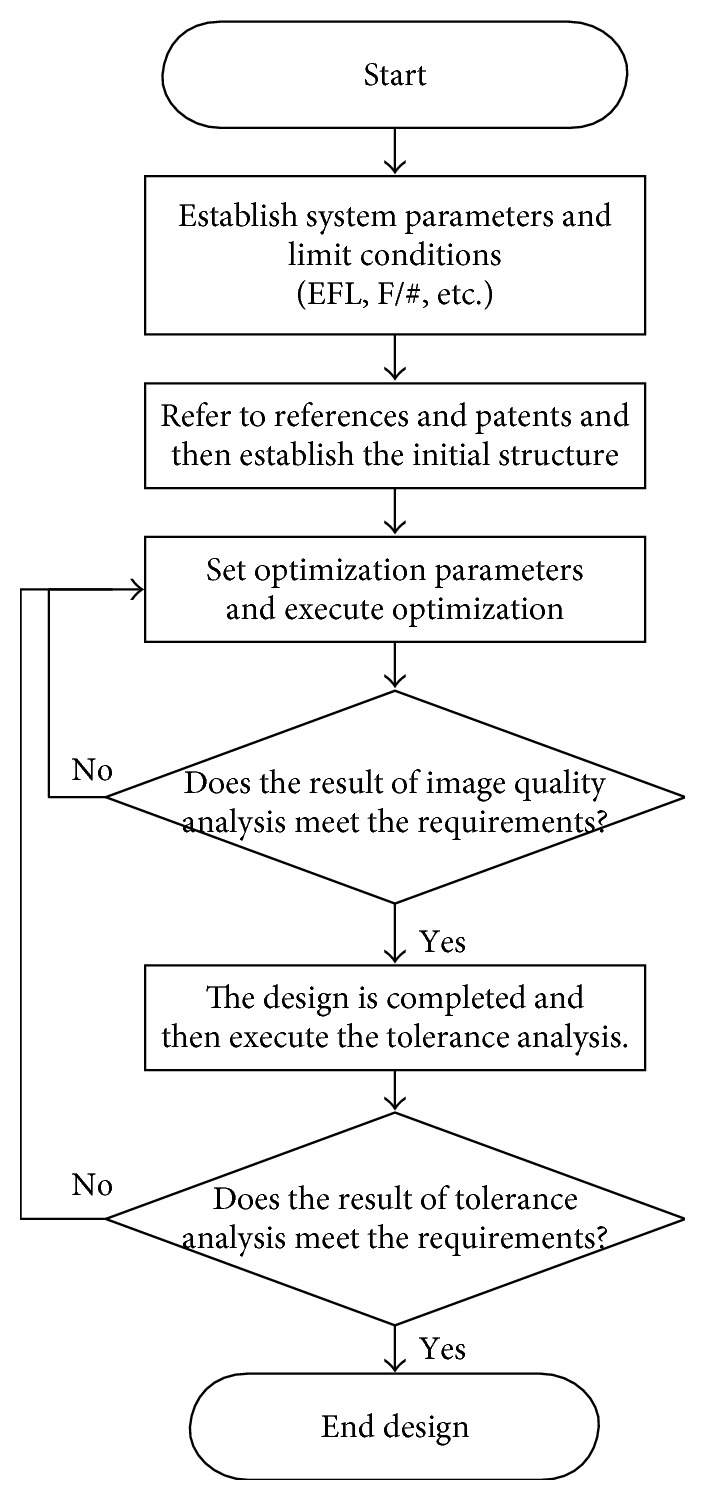
CE design flow chart.

The CE design flow chart of this study is shown in Figure 3.

This section presents the overall design process of the CE optical system; optical system design methods can be divided into the following steps: (1) determining the optical uses of the system, (2) setting design indicators, (3) selecting initial structure, (4) correcting optical aberrations, and (5) testing and inspecting performance.

## 3. Experiment Results

### 3.1. Literature Review

**Figure 4:**
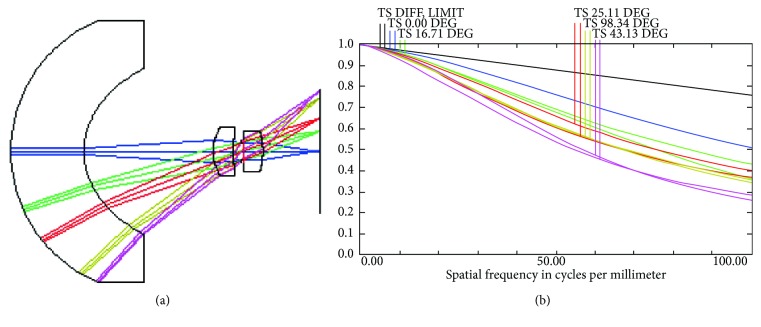
(a) 3D layout used in existent literature. (b) MTF values generated in existent literature.

**Figure 5:**
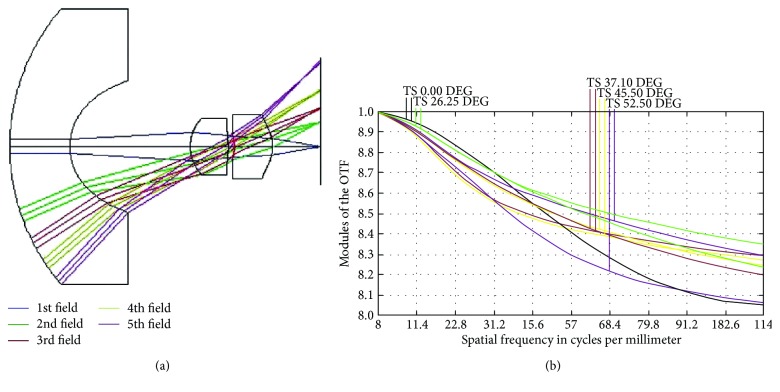
(a) 3D layout used in existent literature. (b) MTF values generated in existent literature.

The CE model used in this study was developed by referring to CE-related literature written by Mang et al. [9] and Tang et al. [10]. Their CE designs are presented as follows:

1. Mang et al. [9]
2. Figure 4(a) shows the camera lens structure presented by Mang et al. [9], which produced an FOV of 86° and an MTF value of 26% at 100 lp/mm (Figure 4(b)).
3. Tang et al. [10]
4. Figure 5(a) shows the design results of Tang et al. [10], which produced an FOV of up to 105°, an MTF value of 40% at 45 lp/mm (Figure 5(b)), and a distortion of less than 15%.

From the 3D layout diagrams of the lenses used in the two studies, we inferred that although both studies adopted 3-lens designs, their MTF values can be further improved. For example, with the advances in imaging technology, CMOS resolutions have been increasing and different lens materials have been introduced. Therefore, in this study, an additional lens was added to the lens group design to enhance image quality (under the constraint of CE size restrictions) and make the lens group applicable to 300-megapixel CMOS designs.

### 3.2. Initial Structural Design

**Figure 6:**
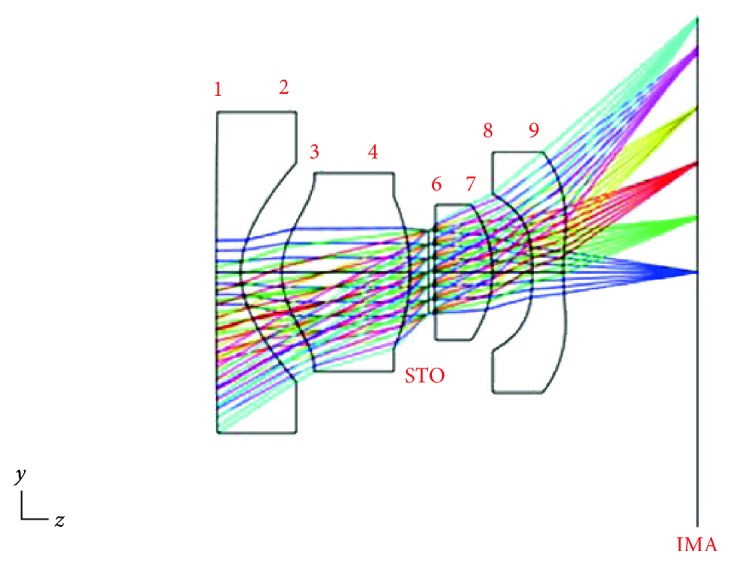
3D image of the initial structure.

The F/# input was set at 2.8. Concerning the FOV, because the CMOS produced an image height of 2.3 mm, real image height was selected for the FOV and inputs 0.0, 0.5, 1.0, 1.5, 2.0, and 2.3 were entered. The wavelengths used were set at 415 and 540 nm considering the use of narrow-band technology.  Table 3 and Figure 6 show the lens parameters and initial structure, respectively, which reveal that the focus of the image plane had a room for improvement.

**Figure 7:**
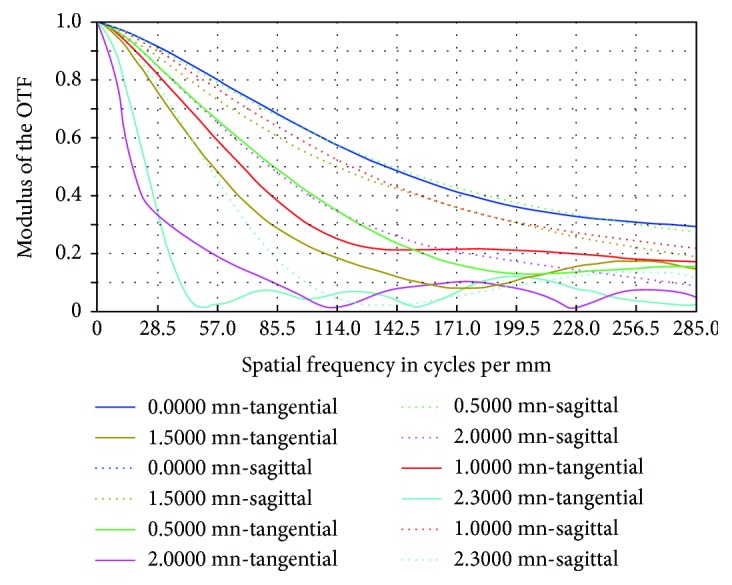
MTF diagram of the initial structure.

Figure 7 shows the MTF diagram of the initial structure, in which the horizontal axis signifies the spatial frequency and the vertical axis denotes how ideal the images were (each image was measured as a percentage of ideal images). The different colored curves represent the MTG under different FOVs. The diagram shows that at the maximum spatial frequency (i.e., 285 lp/mm), MTF was 1.5%.

**Figure 8:**
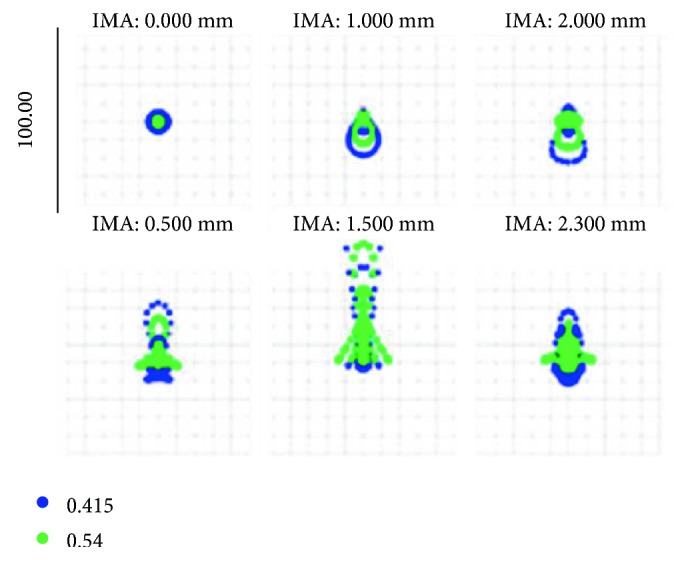
Spot diagram of the initial structure.

The facula diagram shows the magnitude of each light vector on the image plane, as shown in Figure 8. In the diagram, the Airy disk value is 2.161 *μ*m.

**Figure 9:**
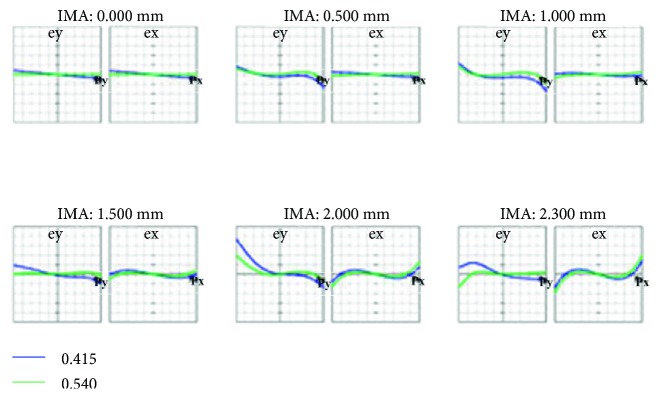
Ray fan diagram of the initial structure.

Figure 9 is the ray fan diagram of each FOV, where the left and right diagrams show the light waves radiated on the tangential and sagittal planes, respectively. Light waves that were farther away from the axis produced more optical aberration, whereas those that were closer to the axis generated less optical aberration.

The addition of the fourth lens changed the overall structure, which increased optical aberration and lowered overall optical system performance. In addition, the optical system had a total length of 4.5 mm, which failed to meet the design objective and had to be further optimized to match design requirements.

### 3.3. Optimized Design

To meet optical design requirements, the initial design structure was optimized using merit functions. ZEMAX design software was employed; it used the damped latest squares algorithm, which involved continuously taking parameter values to diminish the values of the merit functions until the lowest merit function was identified. This method was suitable for parameters that constantly changed. In this study, the merit function was defined as follows:

(5) $$\begin{array}{c} {MF}^{2}=\frac{\sum W_{i}{\left(V_{i}-T_{i}\right)}^{2}}{\sum W_{i}}, \end{array}$$

where *W* is the weight of the operand (in absolute value), *V* is the current value, *T* is the target value, and *i* is the optimization operand number in the merit function table (Table 4).

By using the merit function method, constant optimization was performed until a suitable value was found; the process involved keying in preset operands and their values in the merit function table and complementing it with a built-in operand OPDX (wavefront difference). The optimized initial structure values that were closer to the target values indicated more ideal versions of the camera lens. The optimized lens parameters are shown in Table 5.

**Figure 10:**
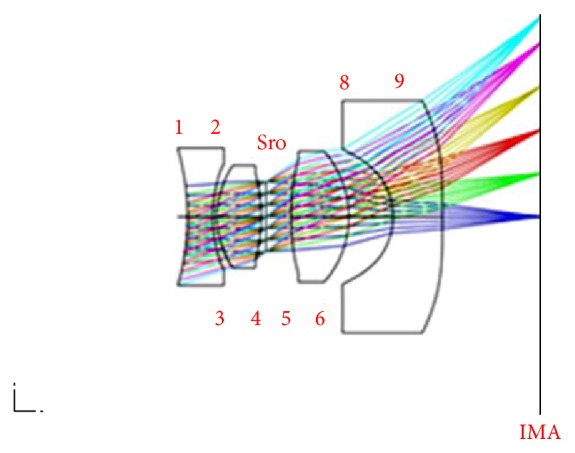
3D image after optimization.

3D images before and after optimization are shown in Figure 10 which shows notable improvements in the focal points on the image plane before and after optimization.

**Figure 11:**
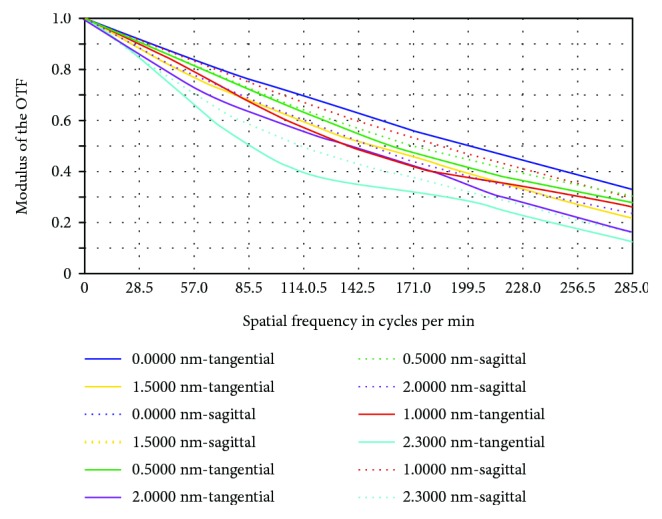
MTF after optimization.

Figure 11 shows that at a maximum FOV of 2.3, MTF increased from 0.015 before optimization to 0.13 after optimization, which is 13% of an ideal image.

**Figure 12:**
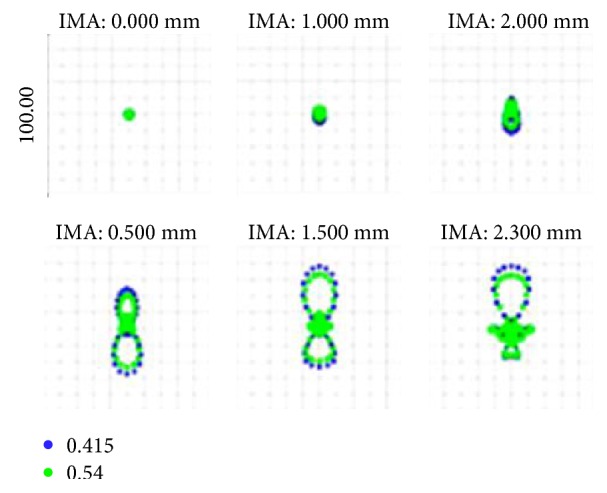
Spot diagram after optimization.

The Airy disk values were 2.161 and 2.584 *μ*m before and after optimization, as shown in the facula diagram in Figure 12.

Table 6 shows the spot RMS radii before and after optimization, which indicated improvements after optimization.

**Figure 13:**
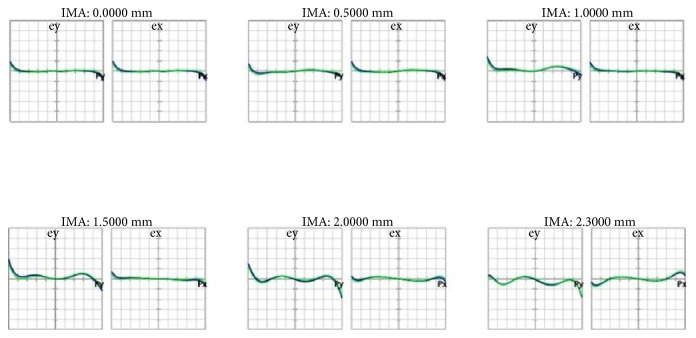
Ray fan diagram after optimization.

Figure 13 shows the ray fan diagram, which was primarily used to analyze optical aberrations. The horizontal axis signifies the relative position of the light ray when exiting the pupil, whereas the vertical axis denotes the position of the light ray (that has left the pupil) on the image plane. Compared with Figure 9, the results improved substantially for the different FOVs.

**Figure 14:**
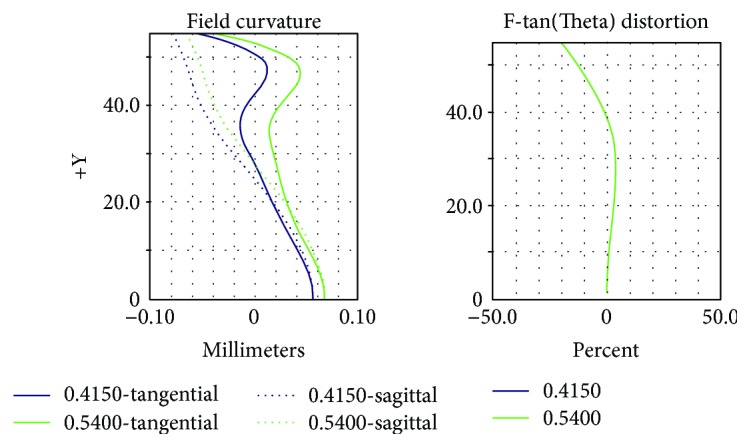
Curvature of the field and distortion after optimization.

Figure 14 shows the curvature of the field, in which the vertical axis represents light waves at different object heights whereas the horizontal axis denotes deviations in the focus positions of paraxial and off-axial rays. The right diagram in Figure 14 is the distortion, in which the vertical axis signifies light waves at different heights whereas the horizontal axis means the differences (measured in percentages) between actual and paraxial rays focused on the image plane. According to the diagram, the size of the distortion was approximately 20%.

**Figure 15:**
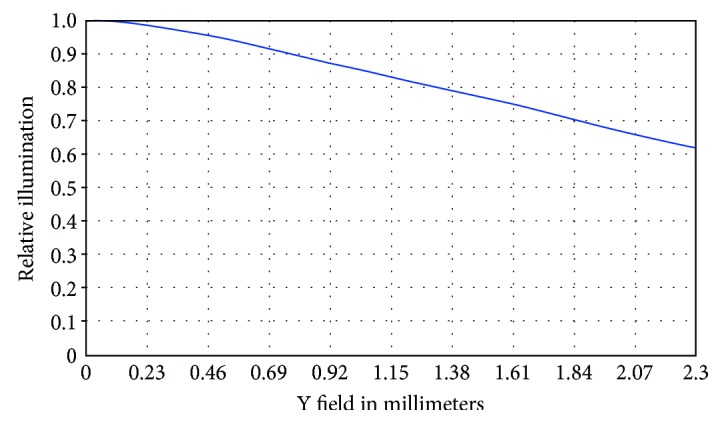
Relative lighting after optimization.

Figure 15 shows the relative lighting graph, which yielded a value of approximately 0.61 (i.e., 61%).

A comparison between results obtained in this study and those presented in [9, 10] is shown in Table 7.

**Figure 16:**
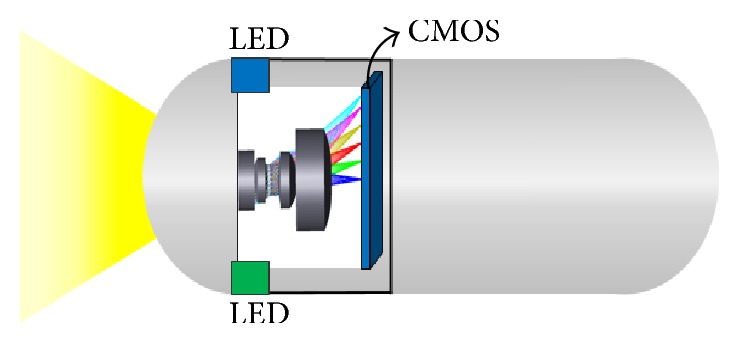
CE mechanism.

Figure 16 shows the optical system schematic diagram of the internal components of a CE. Compared with traditional CEs, the CE introduced in this study adopted an optical lens design that featured the advantages of small volume, low optical aberration, and high resolution. Comparisons with CEs presented in existent literature show that despite the addition of the fourth lens in this study, the total length of the CE remained within size restrictions and that the overall FOV increased. As a popular demand for image quality can be expected to intensify in the future, the CE lens group introduced in this study may serve as a potential option.

## 4. Conclusions

Literature has shown that narrow-band technology has made considerable contributions to the detection of pathological changes in human digestive organs and that such technology has been commonly used in clinical experiments. However, the most common usage of narrow-band technology is in electronic endoscopes, and that in the CE is comparatively scant. Therefore, this study develops optical design technology-incorporated narrow-band wavebands in the CEs based on the lens structure of references [10, 11].

Although the fourth lens was added to the CE in this study, the total length of the CE remained within 4 mm and the FOV (obtained through calculations) was approximately 110°, exemplifying a wider viewing angle that meets the study requirements. In general, the design purposes of CEs are higher resolution and larger FOV angle. There are many researches presenting large FOV angles of CEs, but the resolution is not high enough to let the doctors make diagnoses of the disease easily. In simulation results, the distortion was less than 20% and relative lighting was at least 60%. The MTF values of the CE were 34.1% and 12.5% when the spatial frequency was 144 and 285 lp/mm, respectively. This camera lens design is applicable to CMOS image sensors with a size of 1/4^″^, a pixel size of 1.75 *μ*m, and 3 megapixels. Compared with other CE designs presented in existent literature as shown in Table 7, the CE design introduced in this study displayed superior performance. With the technical advance, the CE image quality can be improved by increasing CMOS resolution and using high-refractive-index material lens in the future.

## Figures and Tables

**Table 1:** Design indicators.

Field of view (FOV)	110°
F/#	1/2.8
Half of the image height	2.3 mm
Effective focal length (EFL)	2 mm
Blake focal length (BFL)	1 mm
Total length	4 mm
Relative illumination	>50%
Distortion	<20%

**Table 2:** Comparison between CMOS selection in this study and that presented in existent literature.

	Ou-Yang Mang	Lilai Tang	Research selection
Active array	256 × 256	1600 × 1200	2048 × 1536
Pixel size	10 *μ*m × 10 *μ*m	2.2 *μ*m × 2.2 *μ*m	1.75 *μ*m × 1.75 *μ*m
Image area	2.7 mm × 2.7 mm	3.6 mm × 2.7 mm	3.6 mm × 2.7 mm
Length of the diagonal line	3.8 mm	2.2 mm	2.26 mm

**Table 3:** Initial lens parameter table.

Surf	Type	Radius (mm)	Thickness (mm)	Glass	Semidiameter (mm)	Conic
OBJ	Standard	15	10		10.143393	0
1	Standard	75.638249	0.228611	E48R	1.467268	0
2	Even Asphere	0.730586	0.375833		0.998383	−0.7580
3	Even Asphere	0.677934	1.181807	CAF2	0.908234	−1.5743
4	Standard	−1.704216	0.173513		0.692586	0
STO	Standard	Infinity	0.055548		0.380945	0
6	Standard	15.517551	0.530639	CAF2	0.441318	0
7	Standard	−1.085120	0.367124		0.619864	0
8	Even Asphere	−3.065604	0.285170	COC	0.714128	14.9694
9	Even Asphere	1.970309	1.236203		1.097346	−43.1582
IMA		Infinity	—		2.328690	0

**Table 4:** Merit function table.

Operand	Meaning	Target
*T*_1_	Effective focal length	2 mm
*T*_2_	Total length	4 mm
*T*_3_	Paraxial image height	2.3 mm
*T*_4_	Relative illumination	55%
*T*_5_	Maximum center thickness of the glass	1 mm
*T*_6_	Minimum center thickness of the glass	0.3 mm
*T*_7_	Maximum edge thickness of the glass	1 mm
*T*_8_	Minimum edge thickness of the glass	0.3 mm
*T*_9_	Distortion max	15%

**Table 5:** Lens parameter table after optimization.

Surf	Type	Radius (mm)	Thickness (mm)	Glass	Semidiameter (mm)	Conic
OBJ	Standard	15	10		8.180505	0
1	Standard	−2.834516	0.278309	E48R	0.750946	0
2	Even Asphere	1.336097	0.031272		0.586826	3.335776
3	Even Asphere	0.749830	0.464941	CAF2	0.564429	−0.887225
4	Standard	−1.847416	0.045157		0.479586	0
STO	Standard	Infinity	0.241786		0.394776	0
6	Standard	2.594178	0.649424	CAF2	0.623751	0
7	Standard	−1.139868	0.489507		0.716711	0
8	Even Asphere	−0.810464	0.628063	COC	0.737654	−4.225100
9	Even Asphere	−3.836154	1.119147		1.327694	−1.203763
IMA		Infinity	—		2.315860	0

**Table 6:** Comparison of RMS radii.

Field	Before optimization	After optimization
0.0	2.555 *μ*m	1.222 *μ*m
0.5	4.946 *μ*m	2.118 *μ*m
1.0	5.713 *μ*m	4.142 *μ*m
1.5	7.769 *μ*m	8.224 *μ*m
2.0	23.756 *μ*m	10.105 *μ*m
2.3	10.064 *μ*m	10.674 *μ*m

**Table 7:** Comparison between results obtained in this study and those presented in existent literature.

	Ou-Yang Mang	Lilai Tang	Result of research
Field of view (FOV)	86°	105°	109.8°
F/#	1/2.8	1/2.8	1/2.8
Total length	<3.5 mm	<4.5 mm	3.94 mm
Relative illumination	43.55%	64%	61.7%
Distortion	<20%	15%	19.14%
MTF (maximum field)	53% (50 lp/mm)	40% (45 lp/mm)	34.1% (145 lp/mm)
	26% (100 lp/mm)	20% (114 lp/mm)	12.5% (285 lp/mm)
CMOS pixels	260 kilopixels	2 megapixels	3 megapixels
